# Editorial: Glucosensing impact on glucose metabolism: From fish to mammals

**DOI:** 10.3389/fendo.2022.1125993

**Published:** 2023-01-06

**Authors:** Xiao-Chen Yuan, Ya-Xiong Tao

**Affiliations:** ^1^ College of Animal Science and Technology, Anhui Agricultural University, Hefei, Anhui, China; ^2^ Department of Anatomy, Physiology and Pharmacology, College of Veterinary Medicine, Auburn University, Auburn, AL, United States

**Keywords:** glucosensing, melanocortin receptor, agouti-related protein, amphibian, fish, mammal

Detecting changes in glucose levels are necessary from fish to mammals to maintain glucose homeostasis and to cope with changes in glucose availability due to dietary habits or environmental conditions. Glucosensing evokes complex neural and endocrine responses to regulate glucose utilization in mammals. Some specialized cells, referred to as glucosensors, detect variations in glucose levels. These cells express various glucose transporters and G protein-coupled receptors (GPCRs) implicated in the physiological response to glucosensing.

Energy homeostasis, including food intake and energy expenditure, are controlled by the arcuate nucleus in the hypothalamus of the central nervous system, consisting of two main subsets of neurons. One subset of neurons express Agouti-related protein (AgRP) and neuropeptide Y, and the other subset of neurons express proopiomelanocortin (POMC) and cocaine and amphetamine-regulated transcript. AgRP is a negative antagonist (inverse agonist) for the neural melanocortin receptors (MCRs), including melanocortin-3 and -4 receptors (MC3R and MC4R), whereas α-melanocyte stimulating hormone derived from post-translational processing of POMC is the agonist for these receptors. The mechanisms of glucose signaling and responses in mammals have been gradually elucidated during the past century. Glucosensing is much less understood in non-mammalian vertebrates. The few studies available suggest that common mechanisms for glucose sensing are present in non-mammalian vertebrates, although more studies are needed to characterize the presence of these systems and to evaluate the functions. In this Research Topic of *Frontiers in Endocrinology*, one review and three research articles pay attention to some aspects of glucose metabolism in fish, amphibian, and mammals.

In the first article, Wang et al. examined the pharmacological and physiological regulation of neural melanocortin signaling, especially Mc3r and Mc4r, in a relatively primitive poikilotherm amphibian species, the Mexican axolotl (*Ambystoma mexicanum*). The melanocortin system consists of five G protein-coupled receptors (MC1R-MC5R), the bidirectional endogenous ligands (MSH and Agouti families) and accessory proteins (melanocortin-2 receptor accessory protein (MARP 1 & MRAP2). A co-expression profile of *mc3r*, *mc4r* and *mrap2* along with *pomc* and *agrp* in the axolotl brain regions and the significant elevation of *mc3r* and *agrp* and reduction of *mrap2* and *pomc* in the hypothalamus upon fasting further confirmed the vital role of melanocortin signaling and their physiological interaction in regulating central feeding behavior and energy balance. The Mrap2 has no effect on the cell surface expression level of Mc3r, but has significant negative effect on the cell surface translocation of Mc4r in a dose-dependent manner. The co-localization and functional complex formation of axolotl MC3R/MC4R with Mrap2 were further confirmed by biochemical and biophysical assays *in vitro*. The pharmacological evaluation of the axolotl central melanocortin signaling reveals its vital physiological role in the regulation of appetite and energy balance in an amphibian species.

In the second article, Zeng et al. developed an *igf1*-deficient zebrafish model using the CRISPR/Cas9 technique to clarify the function of Igf1 on teleost growth regulation. They showed that the Igf1 is not essential for the somatic growth of zebrafish, and other Igfs and insulin can partially compensate for the loss of Igf1. Sexually dimorphic patterns of postnatal growth retardation and metabolic alterations has been observed. More severe defective growth performance associated with clear evidence of fatty liver is observed in *igf1*-deficient males compared to the females. The *igf1* deficiency in zebrafish tends to cause hepatic lipid mobilization defects in males, and glucose metabolism defects in females. This study provide evidence indicating the sexually dimorphic functions of Igf1 for the activation of downstream signaling and glucose uptake in zebrafish.

In the third article, Tai et al. observed the ubiquitous expression of *mrap1* and *mrap2* and the co-expression with *mc1r* transcripts in the skin of diploid amphibian *Xenopus tropicalis*, an animal model for embryonic development and studies of physiological cryptic coloring and environmental adaptiveness. Accumulative studies of vertebrate species highlighted the essential roles of dermal melanocyte-expressed melanocortin 1 receptor (MC1R) on the skin and fur pigmentation, morphological background adaptation, and stress response. This is the first evaluation of the pharmacological profile of Mraps on modulating Mc1r signaling in diploid amphibian species. Co-immunoprecipitation and fluorescent complementation approach further validate the direct functional interaction of Mc1r with Mrap1 or Mrap2 proteins on the plasma membrane. The elevation of the ligand stimulated maximal response of Mc1r signaling suggests that the Mraps participate in the regulation of multiple physiological processes in the skin. The physiological and pharmacological regulations on other GPCR associated pathways in the amphibian species are worthy of future studies.

In the fourth article, Han et al. summarized the latest knowledge on the mechanisms of action and function of AgRP in regulating glucose sensing and metabolism in mammals and fish. The knowledge available in fish about the hypothalamic integration of information about metabolic and endocrine changes in the expression of neuropeptides is limited. This review offers an integrative overview concerning how glucose signals converge on a molecular level in AgRP neurons of the arcuate nucleus of the hypothalamus to control fish food intake and energy homeostasis. Carnivorous fish such as sea bass cannot deal with carbohydrates intake well, with anorexia and metabolic disorders. The dietary carbohydrates converted into glucose signals can be transmitted to the hypothalamus and thereby suppressed the feeding behavior of fish and ultimately limited their growth performance through inhibiting the expression and release of the AgRP neuropeptide within AgRP neurons. Next, the relevant regulatory networks will help solve the efficient utilization of dietary carbohydrates in carnivorous fish.

In summary, the articles present a glimpse of the glucose metabolism in fish, amphibian, and mammals. Some areas of research, for example, the studies on the central glucosensing system, are not included. We hope these articles will stimulate further research on the glucose metabolism from fish to mammals, especially on glucosensing, leading to new and better solutions for metabolic disorders, such as hyperglycemia, glucose intolerance, and insulin resistance.

## Author contributions

All authors listed have made a substantial, direct, and intellectual contribution to the work and approved it for publication.

